# A low-carbohydrate high-fat diet increases weight gain and does not improve glucose tolerance, insulin secretion or β-cell mass in NZO mice

**DOI:** 10.1038/nutd.2016.2

**Published:** 2016-02-15

**Authors:** B J Lamont, M F Waters, S Andrikopoulos

**Affiliations:** 1 Department of Medicine, Austin Hospital, The University of Melbourne, Heidelberg, Victoria, Australia

## Abstract

**Background/Objectives:**

Dietary guidelines for the past 20 years have recommended that dietary fat should be minimized. In contrast, recent studies have suggested that there could be some potential benefits for reducing carbohydrate intake in favor of increased fat. It has also been suggested that low-carbohydrate diets be recommended for people with type 2 diabetes. However, whether such diets can improve glycemic control will likely depend on their ability to improve β-cell function, which has not been studied. The objective of the study was to assess whether a low-carbohydrate and therefore high-fat diet (LCHFD) is beneficial for improving the endogenous insulin secretory response to glucose in prediabetic New Zealand Obese (NZO) mice.

**Methods:**

NZO mice were maintained on either standard rodent chow or an LCHFD from 6 to 15 weeks of age. Body weight, food intake and blood glucose were assessed weekly. Blood glucose and insulin levels were also assessed after fasting and re-feeding and during an oral glucose tolerance test. The capacity of pancreatic β-cells to secrete insulin was assessed *in vivo* with an intravenous glucose tolerance test. β-Cell mass was assessed in histological sections of pancreata collected at the end of the study.

**Results:**

In NZO mice, an LCHFD reduced plasma triglycerides (*P*=0.001) but increased weight gain (*P*<0.0001), adipose tissue mass (*P*=0.0015), high-density lipoprotein cholesterol (*P*=0.044) and exacerbated glucose intolerance (*P*=0.013). Although fasting insulin levels tended to be higher (*P*=0.08), insulin secretory function in LCHFD-fed mice was not improved (*P*=0.93) nor was β-cell mass (*P*=0.75).

**Conclusions:**

An LCHFD is unlikely to be of benefit for preventing the decline in β-cell function associated with the progression of hyperglycemia in type 2 diabetes.

## Introduction

Low-carbohydrate high-fat diets (LCHFDs) have achieved weight loss in several clinical studies,^1, 2, 3, 4^ and others have described their potential benefits in patients with diabetes.^5, 6, 7^ Reducing ingested carbohydrates limits the potential for blood glucose levels to increase following a meal.^6, 8^ However, as the major contributors to hyperglycemia in type 2 diabetes include a resistance to insulin action on target tissues, in combination with an inability of pancreatic β-cells to secrete enough insulin,^9^ it is important we further consider the impact that LCHFD could have on these important aspects of metabolic regulation.

Insulin-stimulated glucose uptake into muscle and adipose tissue is significantly improved by weight loss, such as that achieved in some studies using LCHFDs.^3, 4^ However, an LCHFD does not necessarily result in weight reduction, and high dietary fat in animal studies, regardless of effects on body weight, has been shown to cause an increased accumulation of lipids in the liver, which negatively affects insulin's ability to reduce hepatic glucose production.^10, 11, 12, 13^ Therefore, the proposed benefits vs potential negative effects of an LCHFD on blood glucose control need to be seriously considered before such diets are regarded as a useful option for diabetes management. Moreover, whether LCHFDs will prove beneficial for improving glucose control in type 2 diabetes after long-term use will depend on their impact on glucose-induced insulin secretion from pancreatic β-cells, which prior to this study has not been carefully examined.

Appropriate insulin secretion in response to changes in blood glucose is essential for maintaining normoglycemia. In type 2 diabetes, insulin resistance places a greater demand on pancreatic β-cells to secrete more insulin. If β-cell function is maintained at a level that can compensate for insulin resistance, blood glucose is maintained within the normal range. However, in susceptible individuals, β-cells are unable to cope with the extra stresses of increased metabolism and insulin production that are required. In patients with diabetes, β-cell function is insufficient, and as it continues to decline over time, blood glucose control becomes progressively worse. In this setting, an increase in β-cell apoptosis can also contribute to a loss of β-cell mass.^14^ It is clear that diabetes therapies that do not address this decline in β-cell function fail to maintain blood glucose control.^15^ Therefore, the impacts of LCHFDs on β-cell function may determine whether they could be a useful part of type 2 diabetes management.

A diet or therapy that reduces the workload of pancreatic β-cells in the early stages of diabetes might be predicted to have a beneficial effect for maintaining and perhaps preserving their capacity to respond to increases in blood glucose. In contrast, chronic hypersecretion of insulin has been associated with β-cell failure.^16^ Of potential benefit, reducing dietary carbohydrate reduces postmeal glucose excursions and the need for insulin secretion.^6, 8^ However, in healthy individuals an LCHFD can result in an impaired response to an oral glucose tolerance.^17^ Animal studies are able to shed further light on the potential impact of LCHFDs because they can control dietary and other influences more tightly than clinical studies. HFDs, in combination with either normal or high carbohydrate, have generally induced metabolic impairments in rodents.^10, 18, 19, 20, 21, 22^ Some studies in normal-weight animals have also shown impairments in glucose tolerance and a reduction in β-cell mass.^10, 18^ In contrast, some improvements in glucose homeostasis with an LCHFD were observed in leptin-deficient *ob/ob* mice.^23^ However, none of these studies specifically examined the effect of an LCHFD on β-cell function in an animal model that is prone to diabetes. The New Zealand Obese (NZO) mouse is a polygenic model of obesity, which develops early in the progression of the disease owing to increased energy intake, akin to human obesity.^24^ In NZO mice, which also display glucose intolerance, impaired β-cell function and develop diabetes from approximately 20 weeks of age, a HFD worsened obesity and insulin resistance.^25^ Interestingly, in these mice, a high-fat carbohydrate-free diet prevented hyperglycemia and preserved β-cell mass.^25^ However, when carbohydrate-naive NZO mice were later exposed to a diet containing carbohydrate (32% of energy), they were highly susceptible and quickly developed diabetes.^26, 27^ In summary, a life-long completely carbohydrate-free diet is unlikely to be achievable but an LCHFD, through reducing postmeal glucose excursions, could potentially have some benefit for improving glucose control in diabetes. Therefore, we aimed to determine whether feeding prediabetic NZO mice an LCHFD could positively affect β-cell function and mass. The results presented herein demonstrate that, although postmeal glucose excursions were reduced by an LCHFD, there were no longer-term benefits for β-cell function or glucose metabolism.

## Materials and methods

### Animal diets and housing conditions

NZO mice were bred under specific-pathogen-free conditions in the BioResources Facility at Austin Health (Heidelberg, VIC, Australia). After weaning, male mice were housed in groups of 2–3 per cage and maintained under standard laboratory conditions with controlled temperature (19–22 °C) and 12-h light/dark cycle. All mice were fed *ad libitum* with free access to clean drinking water throughout the duration of this study. Prior to the study, all mice were fed a standard rodent maintenance diet. At 6 weeks of age, mice were either transferred to an LCHFD or maintained on the standard diet (chow) for a further 9 weeks. The LCHFD contained 24 MJ kg^−1^ digestible energy (3.1 MJ or 13% coming from protein, 1.5 MJ or 6% from carbohydrate and 19.5 MJ or 81% from fat (Supplementary Table S1). The carbohydrate content of the LCHFD was exclusively derived from simple sugar (sucrose: 106 g kg^−1^). The fat content of this diet was derived from 55% saturated, 37% monounsaturated and 8% polyunsaturated fats, by weight. The chow diet contained 13.5 MJ kg^−1^ digestible energy, with 2.7 MJ or 20% coming from protein, 9.5 MJ or 70% from carbohydrate and 1.4 MJ or 10% from fat (Supplementary Table S2). Typically, rodent chow carbohydrate is contributed to by 50% starch and approximately 2% simple sugars (monosaccharides plus disaccharides) as a proportion of total carbohydrates by weight. The fat content of chow is typically 18% saturated, 37% monounsaturated and 15.4% polyunsaturated fats. All animal procedures were approved by the Austin Health Animal Ethics Committee.

### Overview of animal experiments

Random-fed blood glucose (at ≈1400 hours), body weight and food intake were measured weekly. Care was taken to account for all the food that was left over and crumbled into the cage. After 6–7 weeks of the diet (at 12–13 weeks of age), mice were fasted overnight and re-fed their respective diets so that insulin and glucose levels could be assessed. Body weight and blood glucose measurements were carried out prior to the removal of food from the cages at 1700 hours. The next day, following the overnight fast (16 h), body weight and blood glucose measurements were repeated, and a 100-μl blood sample was collected from the tail vein. Food was returned, and after 4 h of *ad libitum* re-feeding, blood glucose was measured and another 100 μl of tail blood was collected. After 7–8 weeks, an oral glucose tolerance test (OGTT) was performed as described below. After 9 weeks, an intravenous glucose tolerance test (IVGTT) was performed. Immediately after the IVGTT, animals were killed with a lethal dose of sodium pentobarbital and tissue samples were excised.

### Oral glucose tolerance test

The OGTT was performed in awake mice as previously described.^20, 28^ After administering a standardized glucose dose (37.5 mg in a volume of 150 μl) to all animals, blood glucose was measured at 0, 10, 20, 30, 60, 90 and 120 min, and blood samples were taken at 0, 10 and 30 min. After centrifugation, plasma was stored at −20 °C for future glucose and insulin analyses.

### Intravenous glucose tolerance test

The IVGTT was performed as previously described.^21^ Following the glucose bolus (1 g kg^−1^), blood samples were taken at 0, 2, 5, 10, 15 and 30 min. Plasma was stored at −20 °C for later glucose and insulin analyses.

### Plasma glucose, insulin, triglyceride and cholesterol measurements

A GM7 Analox glucose analyzer (Helena Laboratories, Mount Waverley, VIC, Australia) was used to determine plasma glucose levels via a glucose oxidase assay. Plasma insulin levels were determined using a Mouse Insulin ELISA Kit (Alpco, Caringbah, NSW, Australia). Plasma triglyceride (TRO100) and cholesterol (MAK043) levels were determined using the commercial assay kits (Sigma-Aldrich, Castle Hill, NSW, Australia).

### Tissue extraction and analysis

At the end of the 9-week study, following killing of mice, pancreata and epididymal fat pads were rapidly excised and weighed. Pancreatic tissue was fixed in 10% neutral buffered formalin for 48 h and stored in 70% ethanol until further processing and embedding in paraffin. For assessment of β-cell mass, pancreatic sections were immunostained for insulin (using a guinea pig anti-insulin primary antibody, 1:100 dilution) as previously described.^29^ Two sections (separated by 100 μm) from each pancreas were analyzed. Slides were scanned using the ScanScope CS system (Aperio Technologies, Vista, CA, USA) at × 40 magnification. Digital images were analyzed with the ScanScope software (Aperio Technologies). β-Cell mass was calculated as the product of pancreas weight before fixation and the ratio of insulin positive/total pancreas cross-sectional area.

### Statistical analysis

All data are presented as mean±s.e.m. and *P*<0.05 was deemed significant. GraphPad Prism 6 (GraphPad Software Inc, La Jolla, CA, USA) was used for statistical analysis. Student's *t*-tests were performed to determine statistical significance between the LCHFD and chow groups. When multiple *t*-tests were performed, the Holm–Sidak method was used to correct for multiple comparisons. When data from repeated measures were analyzed, for example, from weekly blood glucose/body weight measurements or GTT curves, or fasting and re-fed conditions, two-way analysis of variance performed and Sidak's multiple comparison test was used to analyse multiple comparisons between the two experimental groups.

## Results

### An LCHFD exacerbates obesity but reduces plasma triglycerides

**Figure 1:**
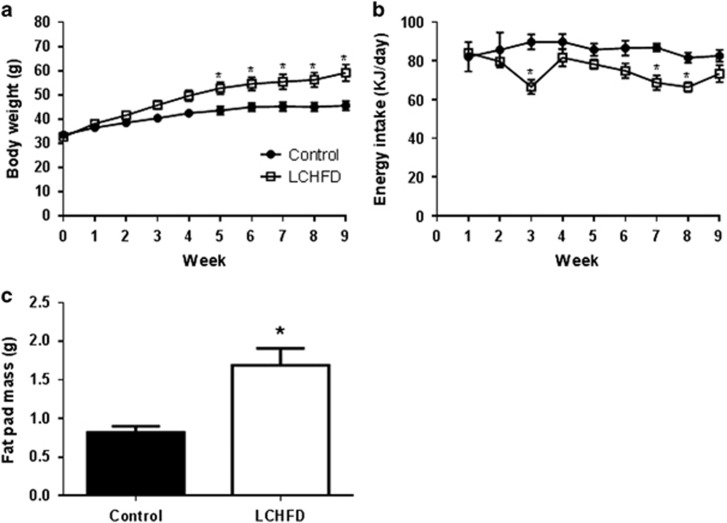
(**a**) Body weight of NZO mice fed either chow or an LCHFD was measured weekly (*n*=9 mice per group). (**b**) Average daily energy intake for NZO mice was calculated from weekly food consumption (for each cage) and the energy content of the respective diets (*n*=5 cages per group). (**c**) Gonadal fat pad weight measured at the end of the 9-week study (*n*=9). Values are presented as mean±s.e.m., *P<0.05 vs chow.

The mean body weight of NZO mice assigned to either the LCHFD (32.6±1.1 g, *n*=9) or standard chow diet (33.5±1.2 g, *n*=8) was not different at the beginning of the study. After 3 weeks, mice fed the LCHFD began to diverge from the chow-fed group, and at 5 weeks the difference in body weight was statistically significant (Figure 1a). At the end of the study, white adipose tissue mass was also significantly increased (Figure 1c). The LCHFD has a higher energy density than the chow diet (24 vs 13.5 MJ kg^−1^); however, the increased body weight of mice fed the LCHFD was not associated with a higher energy intake (Figure 1b).

**Figure 2:**
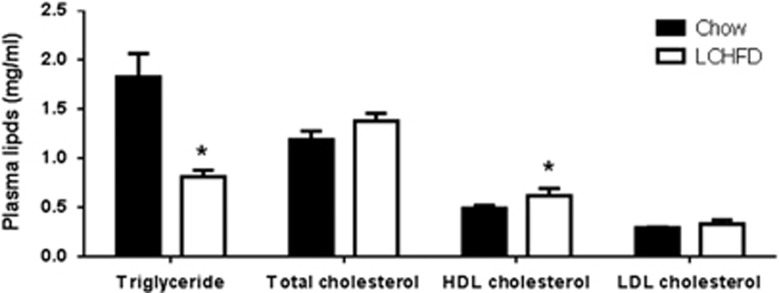
Plasma triglyceride and cholesterol measured in chow and LDHFD-fed NZO mice at the end of the 9-week study (*n*=8–9). Blood samples were collected after overnight fasting. Values are presented as mean±s.e.m., *P<0.05 vs chow.

Despite increased body weight and adipose tissue mass, NZO mice fed an LCHFD displayed reduced plasma triglycerides at the end of the 9-week study (Figure 2). Total cholesterol levels tended to be slightly increased (*P*=0.07) with an LCHFD, and this was mainly due to an increase in high-density lipoprotein (HDL) cholesterol (Figure 2), the predominant form of circulating cholesterol in rodents.

### Increased fasting blood glucose in NZO mice fed an LCHFD

**Figure 3:**
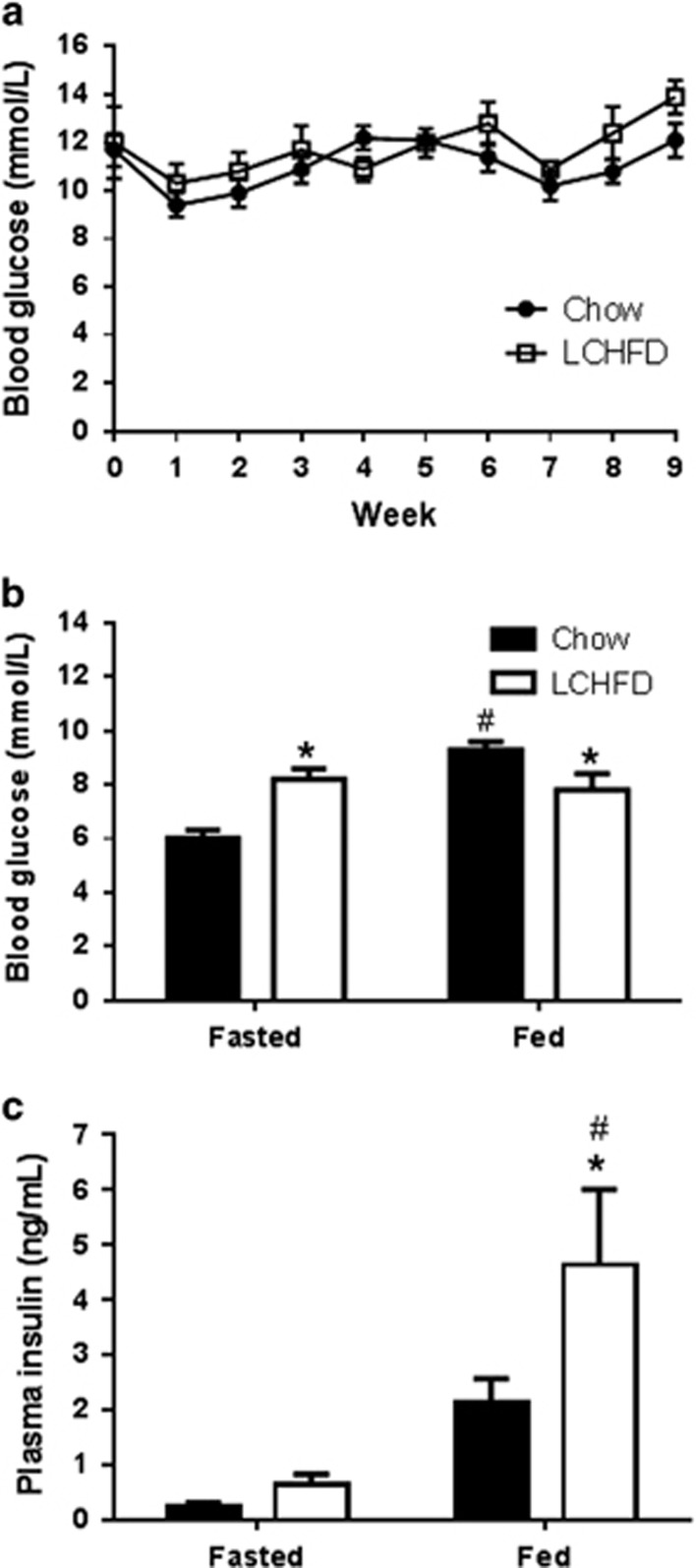
(**a**) Weekly random-fed blood glucose in NZO mice fed either a chow or LCHFD (*n*=9). (**b**) Blood glucose and (**c**) plasma insulin measured after an overnight fast (fasted) and then 4 h after the return of either the chow- or LCHFD-fed mice to the cages (fed). Values are presented as mean±s.e.m. *P<0.05 LCHFD vs control, ^#^*P*<0.05 fed vs fasted (*n*=8–9).

An LCHFD did not affect random-fed blood glucose levels measured weekly throughout the study (Figure 3a); however, the mean blood glucose in the LCHFD group after an overnight fast was significantly higher than that of the chow-fed mice (Figure 3b). There was also a clear trend for higher fasting plasma insulin levels in LCHFD-fed mice (Figure 3c). When mice were given free access to their respective diets after an overnight fast, re-fed blood glucose concentrations were significantly increased following consumption of the carbohydrate-rich chow diet, but they did not change, compared with fasting, in the LCHFD group after feeding (Figure 3b). Therefore, compared with the chow group, re-fed blood glucose in LCHFD-fed mice was approximately 2 mmol l^−1^ lower. Fed plasma insulin levels in the LCHFD mice were also significantly higher (Figure 3c), which is suggestive of exacerbated insulin resistance and consistent with the increased weight gain and adiposity observed in this group (Figure 1).

### An LCHFD further impairs glucose metabolism in NZO mice

**Figure 4:**
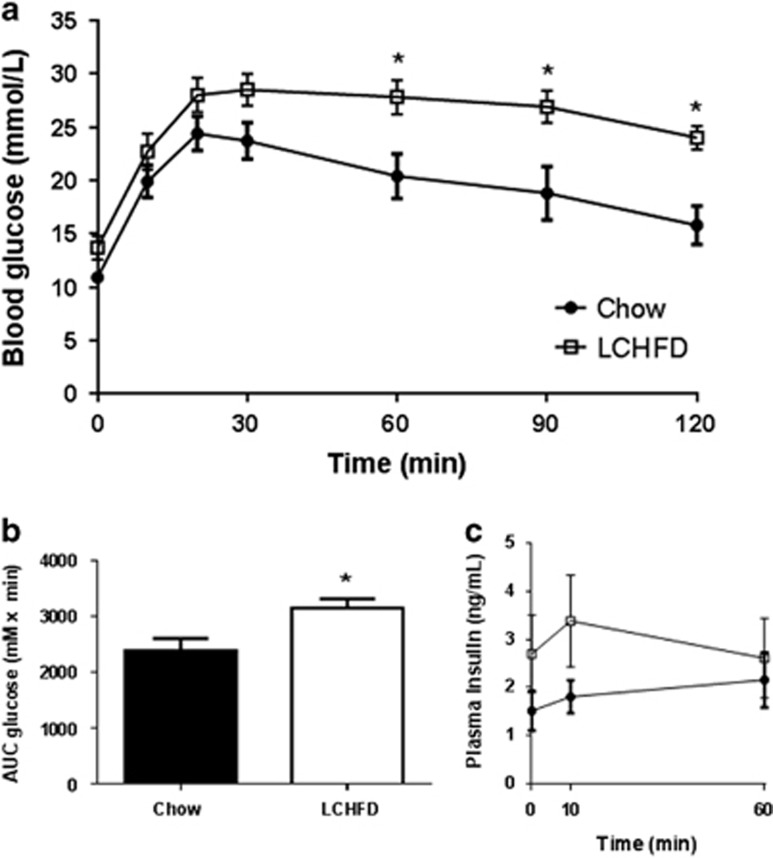
(**a**) Blood glucose at various time points and (**b**) area under the curve (AUC) for glucose after an oral glucose challenge demonstrate that LCHFD-fed NZO mice had impaired glucose tolerance compared with the chow-fed group. (**c**) Plasma insulin was measured in samples collected at 0, 10 and 60 min. Values are presented as mean±s.e.m. *P<0.05 vs control (*n*=9).

In this study, an LCHFD further exacerbated the impairments in glucose metabolism that are characteristic of NZO mice.^30, 31^ During an OGTT, blood glucose levels increased similarly in both LCHFD and chow-fed NZO mice but remained significantly higher throughout the remainder of the test in the LCHFD-fed mice (Figures 4a and b). Although plasma insulin levels also tended to be higher in the LCHFD fed mice (Figure 4c), this was clearly not sufficient to overcome the insulin resistance in these mice.

**Figure 5:**
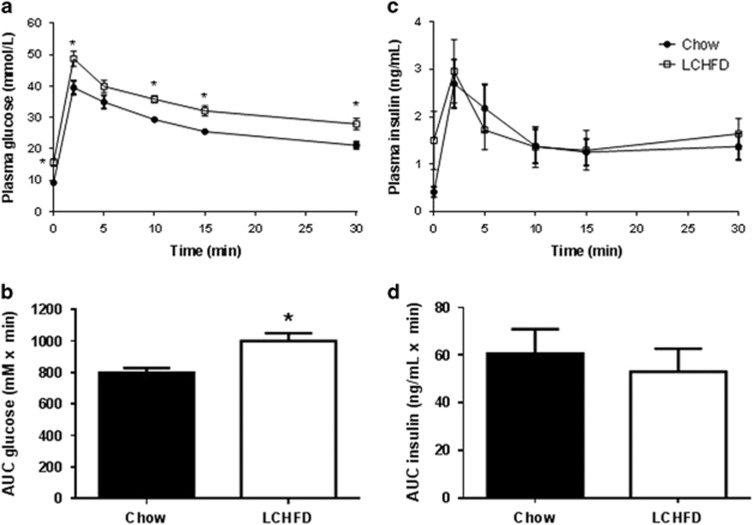
(**a**) Plasma glucose and (**b**) insulin concentrations in chow- and LCHFD-fed NZO mice during an IVGTT. Area under the curve (AUC) for plasma (**c**) glucose and (**d**) insulin were also calculated. Values are presented as mean±s.e.m. *P<0.05 vs control (*n*=8-9).

To further investigate the ability of NZO mice to respond to elevations in blood glucose, an IVGTT was performed. Following an IV glucose bolus, blood glucose levels (Figure 5a) were increased in both groups to levels (>30 mmol l^−1^) that are expected to maximally stimulate insulin secretion from pancreatic β-cells. Overall plasma glucose concentrations during the test were higher in LCHFD-fed mice (Figure 5c), which is consistent with their impaired handling of glucose. Plasma insulin levels in response to IV glucose were increased in both groups of mice, but there were no significant differences between LCHFD and chow-fed mice (Figures 5b and d). Therefore, despite a tendency for higher insulin levels during the OGTT (Figure 4c) and following re-feeding (Figure 3c), this experiment revealed that an LCHFD did not result in an increased capacity to secrete insulin in response to glucose. Indeed, when the incremental area under the curve from the IVGTT was calculated and divided by the 6-h fasting plasma insulin concentration (as a correction for the prevailing insulin resistance), there was a significant decrease in the insulin secretory response (30.5±9.0 vs 9.4±3.5; chow vs LCHFD, *P*=0.03). Therefore, despite limiting postmeal glucose excursions (Figure 3b) an LCHFD has no benefit for β-cell function in NZO mice.

### An LCHFD does not affect pancreatic morphology in NZO mice

**Figure 6:**
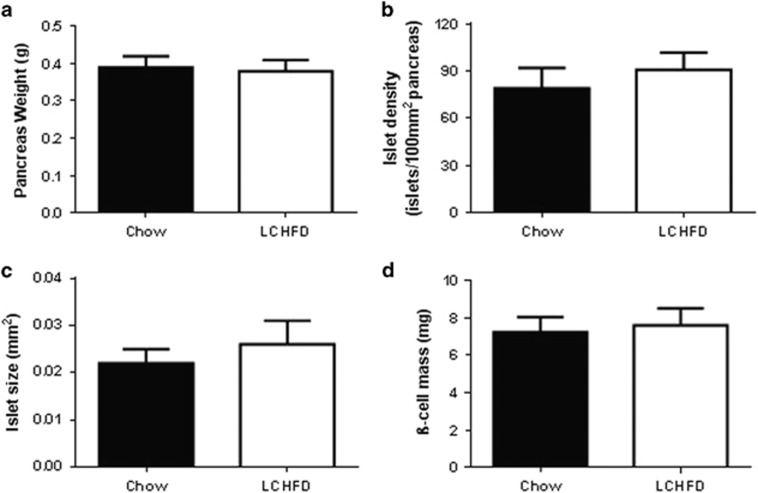
(**a**) Pancreas weight, (**b**) islet density, (**c**) islet size and (**d**) β-cell mass was assessed in the pancreata from NZO mice, collected after 9 weeks of either LCHFD or chow diet. Values are presented as mean±s.e.m. (*n*=7–8 for panels (**a**) and (**d**), *n*=3 for panels (**b**) and (**c**)).

An LCHFD did not affect pancreatic weight (Figure 6a) or morphology. In histological sections, insulin-containing β-cells were identified by immunohistochemistry. Analysis of these sections revealed that islet density, islet size and β-cell mass (Figures 6b–d) were not increased in mice fed an LCHFD. Thus there was no evidence that an LCHFD can have a beneficial effect on pancreatic morphology in NZO mice.

## Discussion

Recently, there has been increased interest as to whether LCHFDs could be of use in battling the growing epidemic of obesity and associated disorders, such as type 2 diabetes. Current dietary guidelines do not define a specific limit of fat intake.^32, 33^ Moreover, a few articles in the lay and scientific literature suggest that the intake of total fat and, in particular, saturated fats may not need to be limited.^5, 34, 35^ For type 2 diabetes, a position statement from the American Diabetes Association recommends that a total fat intake of 20–35% may be desirable for reducing the risk of obesity and suggests minimizing carbohydrate intake is appropriate for improving glucose control, but it has refrained from specifying ideal amounts of macronutrients.^33^ In contrast, some articles are promoting very low carbohydrate intake for diabetes patients, but this approach requires further scientific evidence.^5, 36^ Although LCHFDs reduce postmeal glycemic excursions,^6, 8^ high-dietary fat has clearly been shown, in multiple animal studies, to cause impairments in the ability of insulin to reduce blood glucose resulting in glucose intolerance.^10, 18, 20^ In the current study, we examined the effect of an LCHFD on glucose-stimulated insulin secretion, which is also crucial for maintaining normal blood glucose control.

NZO mice in this study were fed either an LCHFD or standard rodent chow diet. Rodent chow is normally low in fat (3% of energy) and high in carbohydrates (approximately 50% starch). In contrast, the LCHFD used here had a very low carbohydrate (only 6% of energy, 100% sucrose) and high fat content (81% of energy). Most of the dietary fat in the LCHFD was saturated (55% of total fat by weight). This diet therefore models what several papers are recommending for obese or diabetic human subjects.^1, 2, 3, 4, 5, 7^ It has been argued that lowering carbohydrates, whether it is via reduced starch or simple sugars, and replacing it with fat will be beneficial for glycemic control.^5^ In this context, the reduction of dietary carbohydrate in the low-carbohydrate diet studies in humans has been emphasized, without any limitations on the type or source of the fat.^1, 3, 4, 5, 7^ This can lead to an increase in dietary saturated fat intake that is associated with increased blood cholesterol levels,^37^ something that was also seen here in NZO mice fed an LCHFD. As the levels of beneficial HDL cholesterol is also often increased with an LCHFD, there is some controversy as to whether such diets are likely to be associated with adverse cardiovascular outcomes.^5, 37^ Our study was focused on determining the potential effects of this dietary regimen on glucose metabolism.

In prediabetic NZO mice, an LCHFD was accompanied by reduced blood glucose excursion following food ingestion. However, fasting blood glucose and insulin were elevated, suggesting that this diet caused further impairments in insulin action in these obese and insulin-resistant animals. HFDs have been routinely used in normal rodents to induce obesity and metabolic abnormalities, such as insulin resistance.^10, 18, 19, 21^ In NZO mice, our study and others clearly show that increased dietary fat in these genetically obese animals is also associated with greater weight gain and insulin resistance.^25, 27^ In this context, we show that reduced glycemic variability following food ingestion seen with an LCHFD was not associated with any improvement in β-cell function. The increased weight gain in NZO mice following the LCHFD was not associated with increased energy intake and is therefore likely due to reduced energy expenditure, as we have previously shown for mice fed a HFD.^38^ Previous studies in NZO mice have shown that if carbohydrates are completely removed from the diet, hyperglycemia and progression to diabetes can be avoided, but only while the mice remain carbohydrate-free.^25, 26^ However, outside of a tightly controlled animal study, achieving and maintaining a diet that has absolutely no carbohydrates will be practically impossible. In previous studies, when NZO mice were later exposed to dietary carbohydrates, diabetes developed rapidly and was perhaps even more severe than what was observed in mice maintained on a normal rodent chow diet.^26^ In our study, when the LCHFD-fed NZO mice were challenged with an oral glucose load, their ability to restore blood glucose to normal levels was significantly impaired. This further impairment in glucose metabolism in prediabetic mice could be related to the likely impact of increased weight gain on insulin action in the LCHFD group, but such an obvious deleterious effect on blood glucose control can only occur in the absence of β-cell compensation to secrete even more insulin.

A decline in β-cell function is primarily responsible for progressive worsening of blood glucose control in type 2 diabetes.^15^ Improvements in insulin sensitivity that are seen after weight loss and exercise interventions in subjects with impaired glucose tolerance or newly diagnosed diabetes can effect a temporary improvement in β-cell function and reduce hyperglycemia.^39, 40^ Indeed both the Diabetes Prevention Program and the China Da Qing Diabetes Prevention Study showed that lifestyle interventions can significantly reduce the incidence of diabetes in a high-risk population.^41, 42^ Furthermore, caloric restriction has been shown to significantly improve β-cell function in patients with type 2 diabetes.^43^ The early use of aggressive antihyperglycemic pharmacotherapy can also improve β-cell function, resulting in apparent diabetes remission, at least for a period of time.^44, 45^ Therefore, reducing the requirement for insulin secretion appears to have significant benefits for pancreatic β-cells. We have previously shown that high-fat feeding of lean mouse strains led to increased weight gain, hyperinsulinemia and glucose-mediated insulin secretion but not an expansion of islet area.^21, 46^ Here we have shown that an LCHFD reduced glycemic variability after a meal, but it was not able to improve β-cell function or mass in prediabetic NZO mice. Any potential benefit of reducing postmeal glucose excursion with such a dietary approach therefore appears to be outweighed by increased weight gain and a requirement for fasting hyperinsulinemia, which may be detrimental to β-cell function. Indeed, there is mounting evidence that initial hypersecretion of insulin in prediabetes contributes to β-cell stress and failure.^47, 48^ Therefore, rather than being beneficial, an LCHFD may ultimately contribute to faster decline in β-cell function.

Aside from effects on blood glucose levels, LCHFDs have also been purported to have other potential benefits in obese subjects. In NZO mice, an LCHFD was associated with reduced plasma triglycerides, which is consistent with clinical studies that show reducing dietary carbohydrate, even in the context of a high-fat intake, can improve blood lipid profiles.^2, 6, 49, 50^ However, HFDs in both rodent and clinical studies are also associated with higher total cholesterol levels.^2, 18^ In our study, in NZO mice, increased total cholesterol levels associated with the LCHFD, which is high in saturated fat, was mainly owing to increased HDL cholesterol, the predominant form of circulating cholesterol in rodents.^51^ Increased HDL cholesterol is associated with better cardiovascular outcomes^52^ and has been seen with increased dietary saturated fat intake in humans.^53^ However, in the context of other factors associated with high saturated fat intake, whether this has a net benefit for preventing heart failure is not clear. It is therefore important to weigh up all the potential positive and negative effects when considering an LCHFD in patients with metabolic syndrome or prediabetes. If there is increased weight gain and insulin resistance associated with these potentially positive changes in lipid profiles, then it is unlikely that an LCHFD would be beneficial overall.

The potential effect of popular weight loss diets needs to be carefully considered with the help of sound evidence before they are recommended for type 2 diabetes. In our study, we were specifically interested in determining whether an LCHFD would improve β-cell function and therefore whether it may be useful for preventing deterioration in glucose control in a model of obesity and type 2 diabetes. We found that such a diet in prediabetic mice was associated with reduced glycemic excursion after a meal but caused increased weight gain and adipose tissue mass. Increased weight gain requires a chronic hypersecretion of insulin and a greater response to a glucose challenge, but with an LCHFD, there was no improvement in β-cell function or mass in NZO mice. Overall, this diet resulted in greater impairment in glucose tolerance. Our results do not support the recommendation of an LCHFD for use in prediabetes; rather interventions aimed specifically at reducing obesity and improving insulin sensitivity should be pursued.

## Notes

SA receives salary support from an NHMRC fellowship; BJL receives salary support from an NHRMC project grant. The other author declares no conflict of interest.
