# Supplementary material for: A low-carbohydrate high-fat diet increases weight gain and does not improve glucose tolerance, insulin secretion or β-cell mass in NZO mice
Source: Nutr Diabetes. 2016 Feb 15;6(2):e194–. doi: 10.1038/nutd.2016.2 (PMC4775822; doi:10.1038/nutd.2016.2)
Supplement: Supplementary Tables [file nutd20162x1.doc]

**SUPPLEMENTAL TABLE 1**

Low-carbohydrate high-fat diet

| **Ingredient** |  |
| --- | --- |
|  | ***g/kg*** |
| Cocoa Butter | 400 |
| Casein | 200 |
| Sucrose | 106 |
| Canola oil | 100 |
| Clarified butter fat (Ghee) | 100 |
| Cellulose | 50 |
| Calcium carbonate | 13.1 |
| AIN93G vitamins | 10 |
| Potassium dihydrogen phosphate | 6.9 |
| DL-Methionine | 3 |
| Sodium chloride | 2.6 |
| Potassium citrate | 2.5 |
| Choline chloride (75%) | 2.5 |
| Potassium sulfate | 1.6 |
| AIN93G trace minerals | 1.4 |
|  |  |
|  | ***Calculated values*** |
|  |  |
| Protein, *% energy* | 13 |
| Carbohydrate, *% energy* | 6.0 |
| Fat, *% energy* | 81 |
| Digestible energy, *MJ/kg* | 24 |

**SUPPLEMENTAL TABLE 2**

Standard chow diet

| **Ingredients*** |  |  | **Added Vitamins and Trace Minerals** |  |
| --- | --- | --- | --- | --- |
|  | ***g/kg*** |  |  | ***IU/g*** |
| Lysine | 115 |  | Vitamin A | 15 |
| Linoleic Acid | 9.8 |  | Vitamin D3 | 2 |
| Calcium | 9 |  |  | ***mg/kg*** |
| Potassium | 7.6 |  | Vitamin E | 260 |
| Chloride | 4.7 |  | Vitamin K3 | 55 |
| Methionine | 4 |  | Vitamin B1 | 64 |
| Phosphorous | 3 |  | Vitamin B2 | 48 |
| Sodium | 2.5 |  | Vitamin B6 | 30 |
| Magnesium | 1.7 |  | Vitamin B12 | 0.08 |
|  |  |  | Niacin | 400 |
|  |  |  | Panto | 220 |
|  |  |  | Biotin | 1.48 |
|  |  |  | Folic | 11 |
|  |  |  | Iron | 51 |
|  |  |  | Zinc | 60 |
|  |  |  | Manganese | 120 |
|  | ***Calculated values*** |  | Copper | 10 |
|  |  |  | Selenium | 0.1 |
| Protein, *% energy* | 20 |  | Molybdenum | 0.4 |
| Carbohydrate, *% energy* | 70 |  | Cobalt | 0.6 |
| Fat, *% energy* | 10 |  | Iodine | 1.4 |
| Digestible energy, *MJ/kg* | 13.5 |  |  |  |

*data from a typical analysis, raw ingredients for the chow diet included: wheat, wheat byproducts, fish meal, tallow/vegetable oil blend, soybean meal, skim milk powder, yeast, molasses, limestone, salt, vitamins, and trace minerals (as listed in the table).
